# Needlescopic surgery for large umbilical hernia in a patient with morbid obesity using intraperitoneal onlay mesh with fascial defect closure: a case report

**DOI:** 10.1186/s40792-020-01005-6

**Published:** 2020-10-01

**Authors:** Hisataka Fujiwara, Takayuki Suto, Akira Umemura, Yota Tanahashi, Satoshi Amano, Kenichiro Ikeda, Kazuho Harada, Akira Sasaki

**Affiliations:** 1 Surgery Division, Morioka Municipal Hospital, 5-15-1 Motomiya, Morioka, Iwate 020-0866 Japan; 2 Department of Surgery, School of Medicine, Iwate Medical University, 1-1-1 Idaidori, Yahaba-cho, Iwate, 028-3695 Japan; 3 Ikeda-Iin, 5-18 Sakaida, Morioka, Iwate 020-0041 Japan; 4 Anesthesia Division, Morioka Municipal Hospital, 5-15-1 Motomiya, Morioka, Iwate 020-0866 Japan

**Keywords:** Umbilical hernia, Reduced-port surgery, Laparoscopic surgery, Needlescopic surgery, Intraperitoneal onlay mesh, IPOM plus, Obesity

## Abstract

**Background:**

The European and American guidelines recommend that symptomatic umbilical hernias (UHs) are repaired using an open approach with a preperitoneal flat mesh. However, the standard treatment procedure for large UH in patients with extreme obesity is yet to be established. Here, we present the first case of a patient with morbid obesity undergoing laparoscopic UH repair using needlescopic instruments and an intraperitoneal onlay mesh plus repair (IPOM plus).

**Case presentation:**

A 29-year-old man, who was classified as morbidly obese (body mass index, 36.7 kg/m^2^) noticed a reducible nontender mass in the umbilical region and was subsequently diagnosed with an UH, with a diameter of 4 cm. Laparoscopic IPOM plus repair was planned using a needlescopic method for a large UH in the patient with morbid obesity. A 3-mm rigid laparoscope was mainly used in the procedure. After a 12-mm trocar and two 3-mm trocars were inserted, fascial defect closure was performed using intracorporeal suturing with 0 monofilament polypropylene threads. Then, IPOM was performed laparoscopically using an 11.4-cm round mesh coated with collagen to prevent adhesions. The operative time and blood loss were 57 min and 1 g, respectively. The postoperative course was uneventful.

**Conclusions:**

Reduced-port laparoscopic surgery using needlescopic instruments and an IPOM plus technique is a minimally invasive and convenient combination option for large UH in a patient with morbid obesity.

## Background

Umbilical hernia (UH) in adults is a common condition in noninguinal abdominal wall hernias, and increased body mass index (BMI) is associated with higher prevalence and increased risk of incarceration [1]. UHs are prone to incarceration and continue to enlarge if untreated; thus, prompt repair is advised.

Guidelines for the treatment of umbilical and epigastric hernias from the European Hernia Society (EHS) and Americas Hernia Society (AHS) recommend that symptomatic umbilical and epigastric hernias are repaired using an open approach with a preperitoneal flat mesh [2, 3]. Conversely, the guidelines also provided an official statement on the benefit of laparoscopic repair for larger (> 4 cm in diameter) UHs or patients with increased risk of wound infection [2, 4, 5]. Moreover, intraperitoneal onlay mesh (IPOM) with fascial defect closure (IPOM plus) reinforcement in laparoscopic ventral and incisional hernia repair has been introduced and reported in the “Guidelines for laparoscopic treatment of ventral and incisional abdominal wall hernias” published by the International Endohernia Society (IEHS) in 2014 [6]. Based on the guidelines, a laparoscopic approach using IPOM plus may be considered in the repair of large UHs in patients with obesity.

However, minimally invasive surgery has been characterized by the increased development of smaller operative instruments. Recently, reduced-port surgery has become a focus of minimally invasive laparoscopic surgical approaches and has been developed worldwide for various abdominal procedures [7–9]. Reduced-port surgery can be divided into two techniques: needlescopic and single-incision port laparoscopic surgeries. It is known that a single-incision laparoscopic approach increases the risk of hernia formation and is not a naturally ergonomic technique, especially in patients with severe obesity [10]. Therefore, the conventional laparoscopic UH repair usually requires several ports. The needlescopic technique is safely applicable to laparoscopic hernia repair by minimal dissection without the restriction of operation compared with the single-incision laparoscopic approach.

The present systematic review included only English-language articles in the PubMed database identified using the keywords “umbilical hernia”, a combination of “umbilical hernia” and “laparoscopic surgery”, and a combination of “umbilical hernia” and “reduced-port surgery”. No reports to date have described reduced-port surgery for UH using IPOM plus. To the best of our knowledge, the needlescopic approach using one 12-mm trocar and two 3-mm trocars for UH repair in patient with morbid obesity is extremely rare.

Herein, we report the use of the IPOM plus method combined with reduced-port surgery to safely treat a large UH in a patient with morbid obesity with a BMI of 36.7 kg/m^2^, along with some literature review.

## Case presentation

**Fig. 1:**
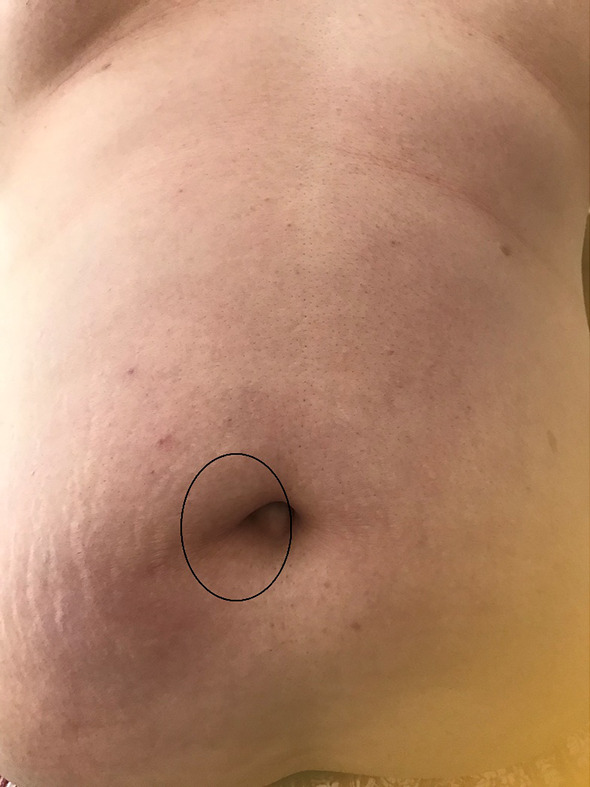
Abdominal photograph of frontal view. A hernia orifice measuring approximately 4 cm is present in the umbilical region (circle)

**Fig. 2:**
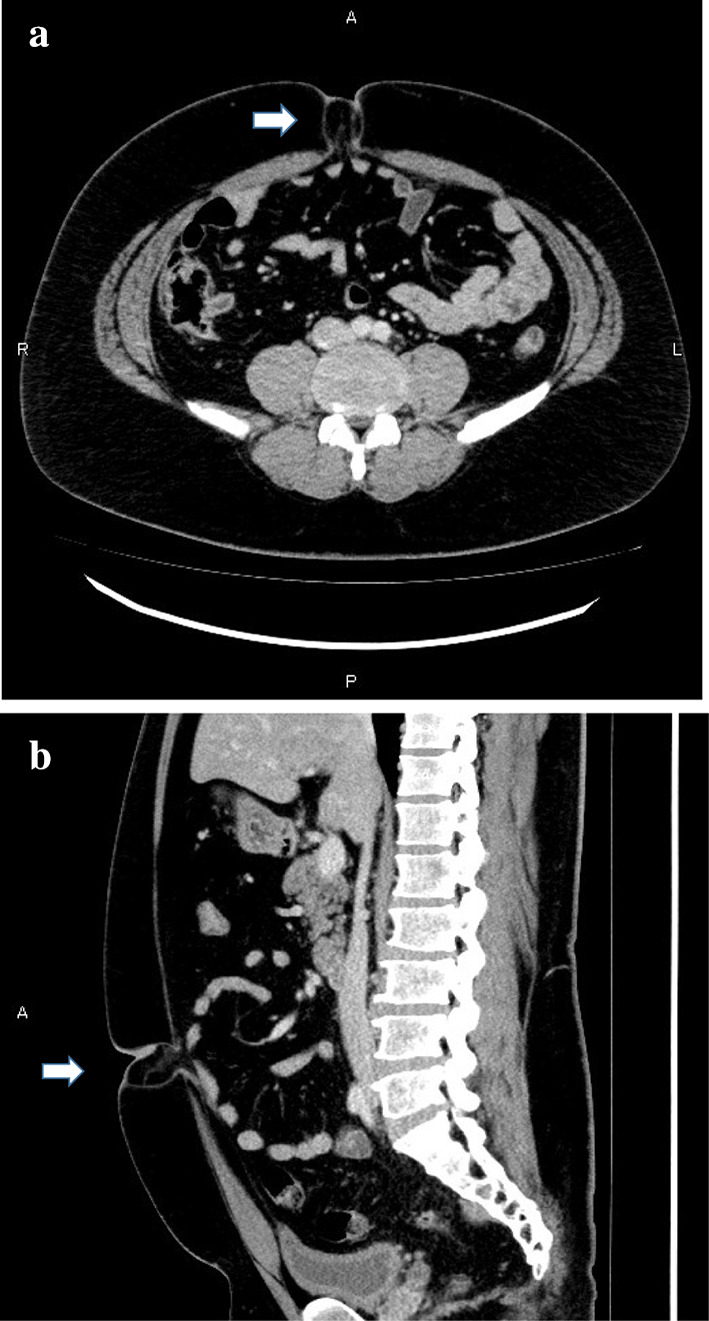
Sections of enhanced abdominal computed tomography. The greater omentum seems to be incarcerated (arrow). **a** Horizontal section. **b** Coronal section

A 29-year-old man was referred to our hospital for further evaluation. He had upper abdominal discomfort, intermittent periumbilical pain, and nausea the previous day. Medical history included morbid obesity (height, 180 cm; weight, 119 kg; BMI, 36.7 kg/m^2^), diabetes, and hyperuricemia. He noticed a reducible nontender mass in the umbilical region a few years before. Physical examination revealed no abdominal distension and tenderness but a visible and palpable mass with an approximately 4-cm hernial orifice in the umbilical region (Fig. 1a). Contrast-enhanced computed tomography also showed UH with part of the greater omentum entering the hernial orifice, but there was no ascites or small bowel obstruction (Fig. 2a, b).

The patient agreed to undergo laparoscopic UH repair. We decided to perform a laparoscopic IPOM plus method to reduce the risk of recurrence and planned to conduct reduced-port laparoscopic surgery using a 12-mm trocar and two 3-mm trocars to minimize the surgical wound.

**Fig. 3:**
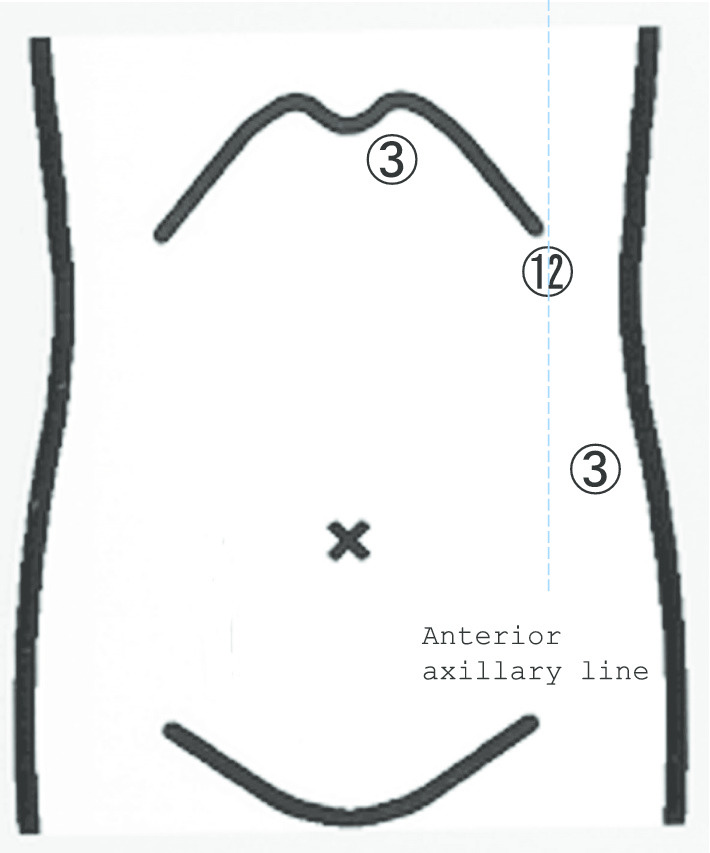
Trocar placement

With the patient in the supine position under general anesthesia, we inserted three trocars (Fig. 3). A 12-mm trocar (ENDOPATH® XCEL; Ethicon, New Brunswick, NJ, USA) was placed on the left hypochondrium region using optical access. After establishing pneumoperitoneum using the insufflation of carbon dioxide up to a pressure of 12 mmHg, two 3-mm trocars were inserted into the epigastric region and left lateral side of the abdomen. A 3-mm rigid laparoscope (KARL STORZ NDTec GmbH, Germany) was mainly inserted into the epigastric region via the 3-mm trocar, and surgery was performed using the other two trocars (12-mm trocar and 3-mm trocar in the left lateral side of the abdomen) as working ports for the operator.

**Fig. 4:**
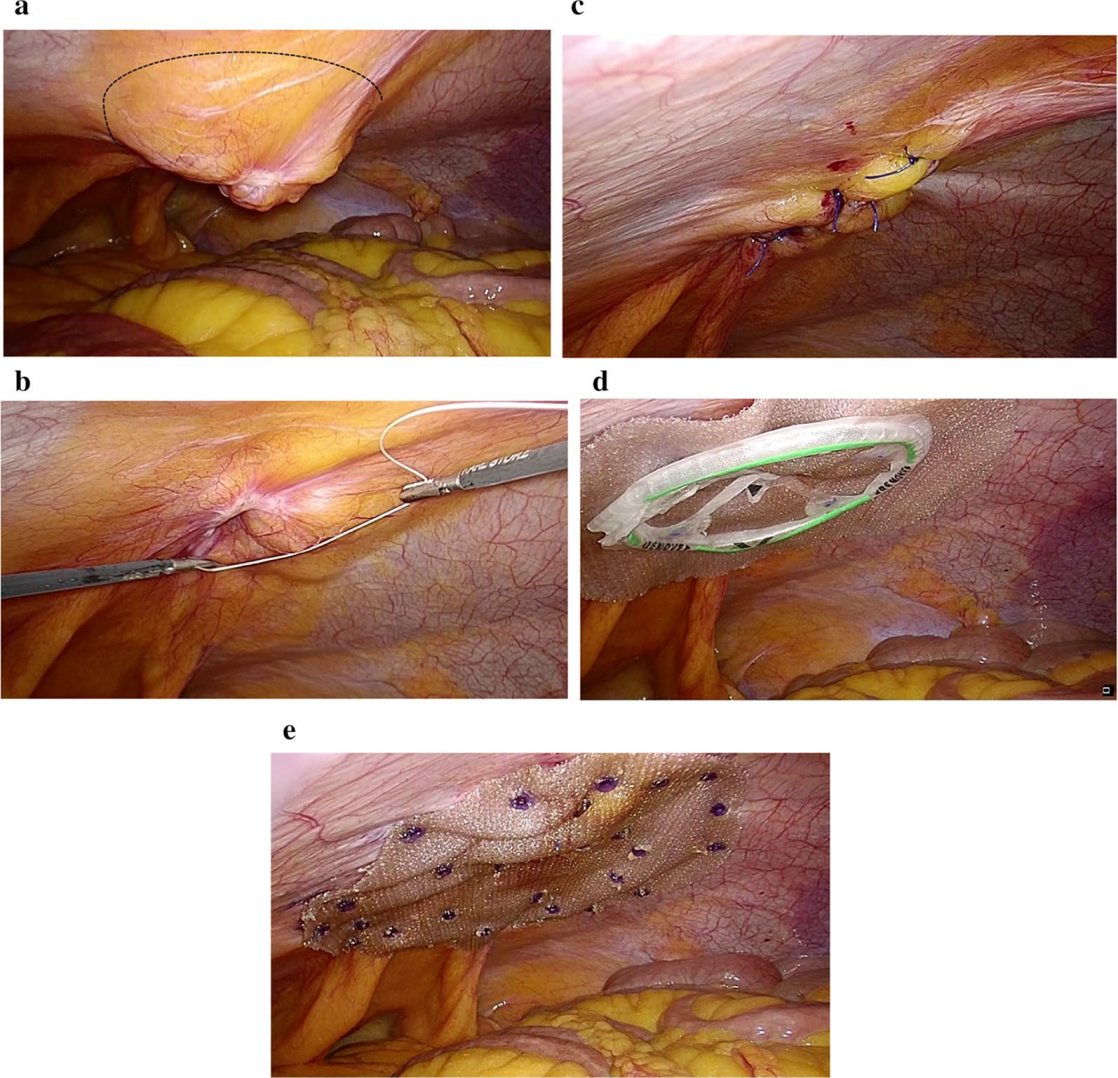
Intracorporeal procedure for umbilical hernia. **a** Palpation of the hernial orifice (circle). **b** Measurement of the hernial orifice. **c** Closure of the hernial orifice with simple interrupted sutures. **d** Temporarily securing the mesh with an attachment to help facilitate deployment. **e** Securing the mesh using the double-crown technique

**Fig. 5:**
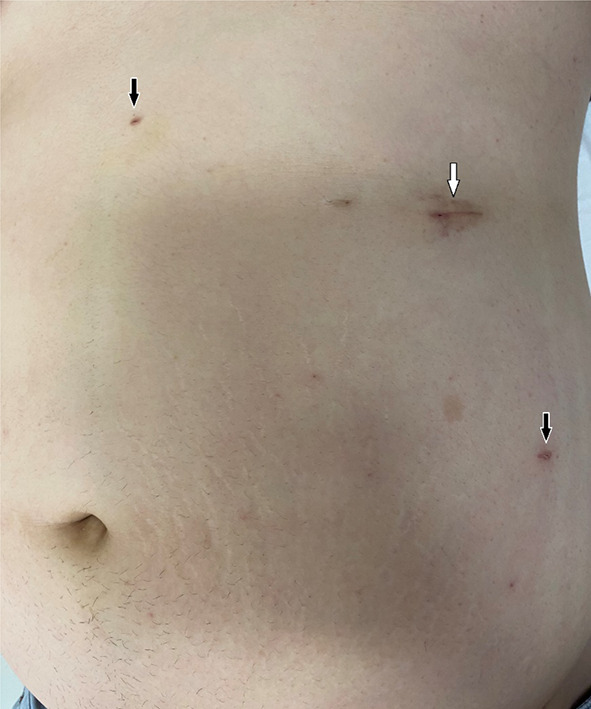
Skin incision wound postoperatively. The insertion site of 12-mm multichannel trocar (white arrow) and insertion sites of 3-mm trocars (black arrows) are obvious

The hernia contents were not observed in the UH, and the margins of the hernial defect were clearly defined under the insufflation pressure of 12 mmHg (Fig. 4a). To prevent an overestimation of the defect size, insufflation pressure was reduced to 6 mmHg at the time of measurement. The hernial orifice was round with a 5-cm diameter (Fig. 4b). First, using the 12-mm trocar as a working port for a 5-mm needle holder, the hernial orifice was closed intracorporeally by simple interrupted sutures with 0 monofilament polypropylene threads (Surgipro™; Medtronic Inc., Minneapolis, MN, USA) under insufflation pressure of 6–8 mmHg (Fig. 4c). Five stitches were required to close the hernial orifice. Then, an 11.4-cm round mesh with an attachment was selected to help facilitate deployment and placement and coated with collagen to prevent adhesions (Ventralight™ ST Mesh with Echo 2™; Bard, Warwick, RI, USA). Next, the mesh was introduced into the abdominal cavity using a 12-mm trocar and placed under the hernial orifice, and the hoisting suture was retrieved through the center of the closure line of the hernial defect and hoisted to the abdominal wall using an EndoClose™ (Medtronic Inc.) (Fig. 4d). An overlap of at least 3- to 5-cm-mesh overlap for the closure line of the hernial orifice was inspected and fixed by a double-crown technique using a mesh fixation device (AbsorbaTack™; Medtronic Inc.) (Fig. 4e). The port-site wound of the inserted 12-mm trocar was closed by two-stitch sutures with absorbable surgical sutures (0 Monosyn®; B. Braun, Melsungen, Germany) using an EndoClose™ (Medtronic Inc.). Moreover, skin incisions were closed by dermal suturing with absorbable surgical sutures. Lidocaine hydrochloride (1%) was inserted into the trocar sites at the end of the surgery. The postoperative wounds are shown in Fig. 5.

The operative time and blood loss were 57 min and 1 g, respectively. The patient received celecoxib 400 mg/day orally for 4 days from the day of the surgery and sometimes needed additional analgesics for postoperative abdominal pain, which is not the port site but mesh fixation region. The postoperative course was uneventful, and the patient was discharged on postoperative day 3.

## Discussion

UHs in adults are common conditions of noninguinal abdominal wall hernias. The current view is that patients with asymptomatic UH and acutely incarcerated UH should undergo surgery to avoid postoperative complications.

Since the introduction by LeBlanc in 1993 [11], laparoscopic ventral and incisional hernia repair has gained popularity globally. Additionally, the recent case series and reviews with long-term follow-up proved that laparoscopic ventral and incisional hernia repair had advantages in terms of lower recurrence and wound infection rates compared with open ventral and incisional hernia repair [2–6].

The guidelines from the EHS and AHS also recommend a laparoscopic mesh-based repair approach for a large UH or in patients with an increased risk of wound infection [2]. Thus, the laparoscopic hernia repair has a great advantage over the open surgery, that is to say, the smaller wound size, the better. Generally, obesity is known to be associated with increased wound morbidity after primary hernia repair [12, 13]. Furthermore, obesity and wound infection increase the recurrence rate. Our patient had morbid obesity (BMI, 36.7 kg/m^2^) and an increased risk of a large hernial orifice (> 4 cm). Therefore, the decision was made to perform a laparoscopic mesh-based repair approach for accurate measurement of the hernial defect size and reduce the risk of surgical site infection. It is also well known that trocar-site hernia was associated with obesity, wound infection, and the use of > 10-mm trocar [14]. Compared with the open access technique, direct trocar insertion was considered superior in terms of port-site hernia, infection, and pain [7, 15]. Thus, we used 3-mm instead of 5-mm trocars mainly. However, we had to select a 12-mm trocar to introduce the mesh, which is bifaceted, polypropylene, and expanded polytetrafluoroethylene, into the abdominal cavity, because few meshes are introduced using < 10-mm trocar. To minimize the surgical wound, we planned to place the 12-mm trocar using visually guided insertion.

Furthermore, we planned to perform reduced-port surgery by placing two 3-mm trocars and using a 3-mm rigid laparoscope. With regard to reduced-port surgery, the single-incision and needlescopic approach has become an increasingly important form of several laparoscopic surgical conditions [8–10], due to the expected benefits of minimally invasive surgery, including less pain and quick recovery from fewer scars and better cosmetic satisfaction. However, the guidelines from the IEHS did not provide an official statement on the benefit of reduced-port technique in the section of key points of the technique [7]. A single-incision laparoscopic approach is an inconvenient ergonomic operation and increases the risk of hernia formation, especially in patients with severe obesity [10]. Meanwhile, a needlescopic approach is a convenient method because it can keep the traditional laparoscopic principle of triangulation. And the 3-mm trocar requires no port-site closure. This point is also beneficial in cosmetic and postoperative pain aspects [16, 17]. Moreover, the incidence of trocar-site hernias is rare in cases with <10-mm trocar sites, especially with 3-mm trocar sites [18, 19]. However, there are some points of caution. The needlescope provides poorer resolution image compared to larger scopes. The small jaws and supple fine shafts of the needlescopic instruments cause difficulties in surgical procedure, especially in cases of severe adhesion or obesity. To avoid the difficulties above, it is important to choose and change the site of the needlescope between the inserted ports according the requirements of the case.

Concerning the IPOM plus technique [7], there are a few high-quality studies on primary ventral hernias focusing specifically on defect closure. Suwa et al. [20] revealed that the recurrence rate, incidence rate of seroma formation, and incidence rate of mesh bulging in the IPOM plus technique for ventral and incisional hernias were 0–7.7%, 0–11.4%, and 0%, respectively. Moreover, Christoffersen et al. [21] reported that the recurrence rate after 2 years and seroma formation after 30 days were 3–17% and 10–33%, respectively. In the absence of abdominal wall tension during fascial closure, an additional fascial closure technique seems to decrease seroma formation, mesh bulging, and recurrence.

In the IPOM plus technique, various fascial closure methods are classified into extracorporeal or intracorporeal suture methods. We have generally selected the intracorporeal suture method, which is a more difficult procedure, because extracorporeal suture methods seem to increase the risk of skin infection by many stabs on the skin. Conversely, if the IPOM plus technique is selected, a landmark for the overlap of the mesh was divided into two groups: according to the border of the original fascial defect or after the closure of the defect by sutures. Based on the guidelines, a large overlap is recommended for large hernias [3]. Suwa et al. [20] revealed that the recurrence rate showed no significant difference between the overlap selected based on the original fascial defect and after closure line. If the mesh size is selected based on the closure line, an overlap of > 5 cm occurs more easily, especially for a transverse diameter. We have considered that a reduction in the recurrence rate could be expected by selecting mesh size based on the closure line.

We have presented the first case of a patient with morbid obesity who underwent reduced-port laparoscopic UH repair employing an IPOM plus technique.

We have considered that employing the IPOM plus technique may potentially reduce the recurrence rate of various umbilical or ventral hernias. Moreover, reduced-port laparoscopic surgery using the needlescopic technique for a patient with morbid obesity decreased the risk of port-site hernia and surgical site infection without affecting the safety or quality of the procedure.

In conclusion, this case has followed a satisfactory course without any troubles, whereas it is obvious that the difficulty level of this surgery depends on the adhesion in the abdominal cavity and the size/position of the hernia orifice. Our successful cases, however, strongly suggests that needlescopic surgery has the potential to play major role on the UH repair in the patients with morbid obesity.

## Data Availability

Not applicable.

## Abbreviations

UH: Umbilical hernia
UHs: Umbilical hernias
EHS: European Hernia Society
AHS: American Hernia Society
IPOM: Intraperitoneal onlay mesh
IPOM plus: Intraperitoneal onlay mesh with fascial defect closure
IEHS: International Endohernia Society
BMI: Body mass index
